# Traumatic Coronary Artery Dissection with Secondary Acute Myocardial Infarction after Blunt Thoracic Trauma

**DOI:** 10.5334/jbr-btr.1379

**Published:** 2018-01-04

**Authors:** Gert-Jan Allemeersch, Caroline Muylaert, Koenraad Nieboer

**Affiliations:** 1 UZ Brussel, BE

**Keywords:** traumatic corornary artery dissection, RCA, AMI, blunt chest trauma

## Abstract

We report the case of a 41-year-old male with traumatic coronary artery dissection after a high-speed motor vehicle collision. Computed tomography imaging revealed multiple intracranial subdural and subarachnoid bleedings, a skull base fracture and multiple bilateral rib fractures. There was no pericardial hemorrhage, haemothorax or pneumothorax. No intra-abdominal lesions were found. A 12-lead electrocardiogram on arrival showed an acute myocardial infarction. Emergency angiography showed complete dissection of the right coronary artery without reflow after placement of 6 coronary stents. The patient passed away the day after. In retrospective, the right coronary dissection was visible on the trauma CT-scan.

## Introduction

Blunt chest trauma can cause severe thoracic injuries like pneumothorax, lung contusion or haemothorax, but can also involve the heart, including the myocardium, pericardium, the large thoracic vessels and the coronary arteries. Of these, coronary artery dissection with secondary acute myocardial infarction is a rare event. Early recognition is critical for survival.

## Case Report

**Figure 1:**
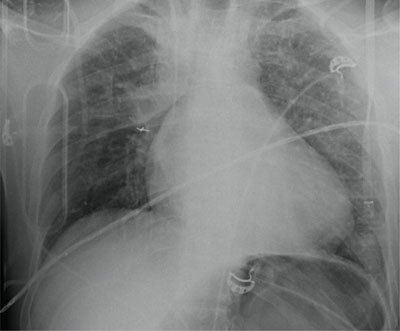
Multiple left sided rib fractures without pneumothorax and blurry consolidations in both lungs, probably lung contusions.

A 41-year-old man was admitted to the emergency department (ED) after a high-velocity car accident. He was ejected out of his vehicle. He had a severe bleeding head wound, symmetric breathing, and normal abdominal findings. Blood pressure and saturation were normal. On arrival to the hospital the patient had worsening bradypnea and bilateral rhonchi. A chest X-ray (Figure 1) shows multiple left sided rib fractures without pneumothorax and blurry consolidations in both lungs, probably lung contusions.

After stabilization of the patient, he immediately underwent a computed tomography (CT) of the head, followed by a total body CT from the circle of Willis up to the pelvis with a split bolus intravenous contrast injection. This study shows multiple intracranial subdural and subarachnoid bleedings with secondary edema of the left hemisphere and a skull base fracture.

Multiple rib fractures are seen on the left side, without a notion of a flail chest, with underlying lung laceration on the left side and contusion. There are no signs of a haemothorax or a hemopericardium, nor an intra-abdominal bleeding or a laceration of the visceral organs are observed on CT.

**Figure 2:**
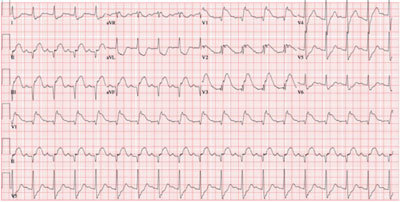
ECG on admission shows sinus tachycardia (109 bpm) and the presence of an ST segment elevation inferior. The ST elevation in lead III is more pronounced than in lead II, which suggests an occlusion of the RCA. There is also an ST elevation in leads V1–V3, with more important ST elevation in lead V1 than V2, suggestive of concomitant right ventricular infarction.

**Figure 3:**
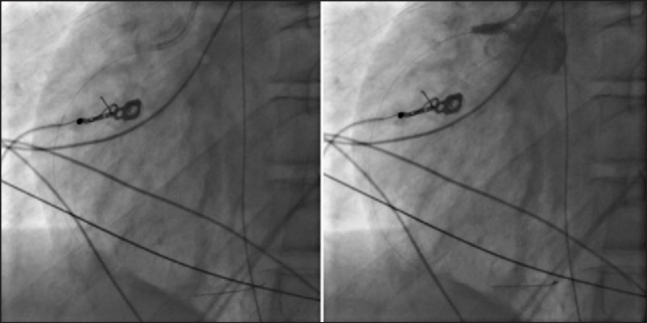
Coronary angiography. Left side image showing guide in the RCA. On the right-side image during injection of iodized contrast showing backflow in the aorta and no enhancement of the RCA compatible with complete occlusion.

**Figure 4:**
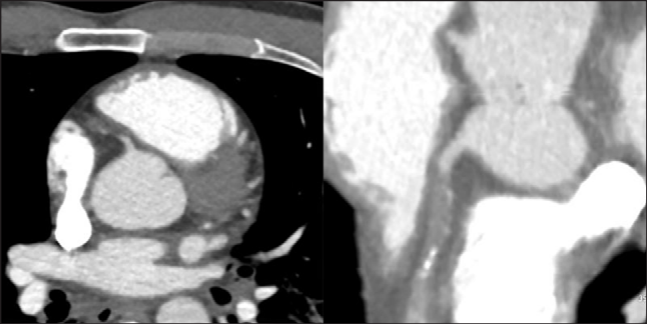
Spiral acquisition trauma CT of the thorax and abdomen after intravenous injection of 100 ml iodized contrast. Focus on the heart showing dissection and complete occlusion of the RCA. Left image: axial view, right image: curved view.

A 12-lead ECG (Figure 2) demonstrates an acute myocardial infarction. An urgent angiography was performed (Figure 3). The angiography shows a complete occlusion of the right coronary artery (RCA), probably due to a traumatic dissection. Multiple stents were placed to approve reflow of the myocardium. There was no reflow to the myocardial tissue established, and the patient, unfortunately passed away. On review of the trauma CT, the dissection of the RCA was visualized (Figure 4).

## Discussion

A clinically significant cardiac injury occurs in 5% to 15% of patients with severe blunt chest trauma [1,2,3,4]. Coronary artery dissection has a very low incidence. Coronary artery dissection due to blunt chest trauma is even less common and a life-threatening complication [5]. A traumatic dissection is most commonly seen in the LAD (76%), followed by the RCA (12%) and circumflex artery (6%) [6]. The mechanism is poorly understood, but the vulnerable anatomic position of the heart is probably the explanation why the LAD is most affected. The RCA is likely to be most vulnerable at its origin due to acceleration/deceleration injuries [7].

Christensen et al. reviewed 76 published cases of post-traumatic myocardial infarctions [8]. In almost 90% of the cases, the myocardial infarctions were due to motor vehicle collisions or high-velocity trauma. In about 70% of these cases, the infarction was caused by occlusion or dissection of a coronary artery. The presentation or recognition of a traumatic coronary artery dissection can be very difficult. Dissection of the coronary artery can be completely asymptomatic or result in acute coronary syndrome and sudden death [9].

The presentation could be delayed; these are probably the patients seen in emergency departments. Probably the incidence of post traumatic artery dissection is much higher but is underdiagnosed since several patients do not make it to an ED fast enough and die at the accident scene or on their way to the ED. Early detection of the coronary artery dissections is thus crucial to reduce mortality. This diagnosis requires clinical suspicion and is based on ECG. Hyper acute T-waves are seen on electrocardiography in the affected territory, followed by ST-segment elevation. If this is the case, then coronary angiography is the primary diagnostic imaging modality. Post-traumatic coronary disorders detected with ECG-gated CT coronary angiography have been described [10].

In this case report, the patient first underwent a non-ECG gated full body trauma CT where at first the coronary dissection was not seen. After diagnosis of the myocardial infarction with ECG and proven dissection/occlusion of the RCA on coronary angiography we reviewed the CT. In retrospective, the coronary artery dissection was revealed. We found no case reports where a coronary dissection was observed on non-ECG-gated trauma CT. Faster and wide detector CT technology makes trauma CT an imaging tool for detection of coronary dissection even if the scan is non-ECG gated. Especially patients with a slow heart rhythm or bradycardia have a much higher potential for detection of the dissection/occlusion of the coronaries. Radiologists and clinicians should be aware of this and look more peculiar to the coronary arteries when assessing a trauma CT of the chest.

## Conclusion

In the condition of a high-velocity blunt chest trauma without signs of hemopericardium, haemothorax or pneumothorax, but with signs of acute myocardial infarction the clinician/radiologist should look more peculiar for the coronary arteries when evaluating a trauma CT scan of the thorax. The latest CT technology is so fast and accurate that it can capture images of the heart with minor motion artifacts even in non-ECG-gated trauma whole body CT, especially when the patient is bradycardic.
